# Supplementary material for: Chemical and Biological Components of Urban Aerosols in Africa: Current Status and Knowledge Gaps
Source: Int J Environ Res Public Health. 2019 Mar 15;16(6):941. doi: 10.3390/ijerph16060941 (PMC6466367; doi:10.3390/ijerph16060941)
Supplement: Supplementary file 1 [file ijerph-16-00941-s001.pdf]

Table S1: A selection of the studies carried in African cities on ambient particulate matter size fraction. These studies show the ranges of mean PM_{10} and $PM_{2.5}$ levels and methodology used such as site characteristic, sampling device with different flowrate, sampling duration of PM and sampling time.

Reference	Study location	Site characteristic	Sampling device	Flowrate	Sampling duration	Sampling period	$PM_{2.5}$ [μg/m³]	PM_{10} [μg/m³]
Kalisa et al. [1]	Kigali, Rwanda	Traffic	HVAS	1000 LPM	24h	Apr to Jun 2017	185	214
		Urban					81.4	98.7
	Musanze, Rwanda	Rural					45	53.7
Pope et al. [2]	Nairobi, Kenya	Traffic	Low-cost (OPCs)	1m³/h	24h	Feb to March 201	36.6	93.7
		Urban					24.8	53
		Rural					13	19.5
Ouafo-Leumbe et al. [3]	Djougou, Benin	Rural	MVPS	5 LPM	**	Nov 2005 and Oct 2009	0.7 to 47.3	1.4 to 148.3
Léon et al. [4]	Cotonou, Benin	Traffic	PPS	10 LPM	12h	Jan 6 to 11 and July 5 to 10 2016	335.1	*
Djossou et al. [5]	Cotonou, Benin	Traffic	MVPS	5 LPM	15min	Feb 2015 to March 2017	11 to 174	
	Abidjan ,Côte d'Ivoire	Traffic					8 to 226	*
Bouacha [6]	Tiaret, Algeria	Traffic	CI	**	**	May to August 2016	20	37
Terrouche et al. [7]	Constantine, Algeria	Traffic	LVAS	5 LPM	**	23 Dec 2011 and 8 Jan 2013	57.8	105.2

Ediagbonya et al. [8]	Sapele, Nigeria	Industrial	LVAS/MVAS	5 LPM	24h	**	104 to 260.4	104.2 to 431.0
Kirenga et al. [9]	Kampala, Uganda	Traffic	DUSTTRACK	**	24h	30 Jun to 27 Jul 2014	132.2	*
Bahloul et al. [10]	Sfax , Tunisia	Industrial	NFAS	**	24h	Nov 23rd to Dec 16th, 2013	*	4.07 to 88.51
Ana et al. [11]	Ibadan, Nigeria	Urban<br>Traffic<br>Industrial	**	**<br>**<br>**	6h	Jan and March 2008.	*	422<br>328<br>257
De Longueville [12]	Kandi, Benin	Rural	Satellite data	**	**	Feb 2003 and Dec 2007 (61 days)	*	1017
Garrison et al. [13]	Mali, Bamako	Traffic	MVAS	5 LPM	24h	Sept 2012 and Jul 2013	43	210
Lowenthal et al. [14]	Shobra, Egypt	Industrial	LVAS/MVAS	5 LPM	24h	Jun and Oct 2010	61.0 to 216.0	154.0 to 360.0
Val et al. [15]	Bamako , Mali<br>Dakar ,Senegal	Traffic	CI	30 LPM	48h	2008 to 2009	*	205.8<br>80.7
De Longueville et al. [16]	Banizoumbou, Niger<br>Cinzana, Mali<br>M'Bour, Senegal	Rural<br>Rural<br>Rural	TEOM	**	**	2006 to 2007	*	187<br>129<br>108
Dieme et al. [17]	Faidherbe, Senegal<br>Fann, Senegal<br>Ngaparu, Senegal	Traffic<br>Traffic<br>Rural	HVCI	68 m^3/h	15 days	Jul 2009 and Sept 2009,	105.4<br>75.1<br>16.9	*
Kinney et al. [18]	Nairobi, Kenya	Traffic	PFAS	4 LPM	11h	Jul 2009	98.1	*

Worobiec et al. [19]	Bethlehem, South Africa	Urban	Dust-monitor	1.2 LPM	**	Jul-01	1000	*
Dionisio et al. [20]	Accra, Ghana	Traffic<br>Urban	DustTra k	**	48h	Nov 2006 and Aug 2008	39 to 53<br>0 to 70	80 to 108<br>57-106
Gebre et al. [21]	Addis Ababa, Ethiopia	Urban	AP	**	24h	22 Feb to 15 Apr 2008	*	80
Mkoma et al. [22]	Dar es Salaam, Tanzania	Traffic	LVAS	17 LPM	12h	Aug and Sept 2005	26	76
Odhiambo et al. [23]	Nairobi, Kenya	Urban	SFU	18 LPM	8h	Feb - Apr 2003	*	239
Zghaid et al. [24]	Kenitra, Morocco	Traffic	PAS	17LPM	24h	2010, 2011 and 2012 (14 months)	51.32	115.12
Boman et al. [25]	Ouagadougou, Burkina Faso	Traffic	Cyclones	3LPM	24h	Nov -Dec 2007 (12 days)	27	164
Arku et al. [26]	Accra, Ghana	Traffic	HCI	10 LPM	24h	30 Jun to 20 Jul 2006.	27.4	71
Laakso et al. [27]	Bethlehem, South Africa<br>Harare , Zimbabwe	Industrial	MVAS	**	13h	July- to Dec 2002	 40	60<br>60
Efe & Efe [28]	Warri , Nigeria	Traffic	AP	**	24h	Annual 2003		126
Van Vliet et al. [29]	Nairobi, Kenya	Traffic<br>Urban	Cyclone	4LPM	12h	Feb 2016 (4 days)	414<br>20	*
Etyemezian et al. [30]	Addis Ababa, Ethiopia	Traffic<br>Sub -urban	MVAS	5 LPM	24h	Jan -Feb 2004	100<br>40	*

Engelbrecht et al. [31]	Bethlehem, South Africa	Urban	ALVS	16.7 LPM	24h	June -July 1997	109	*

†Particulate matter: PM $_{2.5}$ (particles less than 2.5 μm in diameter), PM_{10} (particles less than 10 μm in diameter) **† Sampling device:** ALVS (Anderson Low Volume Air Sampler), MVAS (Minivol Air Samplers), LVS (Low Volume air sampler), HVCI (high volume cascade impactor), HVS (High volume sampler), PAS (Personal air Samplers), AP (Air pump), HCI (Harvard cascade impactors), OPC (Optical particle counter), PFAS (Personal filter based air Samplers), SFUAS (Stacked Filter Unit air sampler), NFAS (Nucleopore filter based air sampler), PPS (Portable personal sampler), TEOM (Tapered element oscillating microbalance) **†Flowrate:** LPM (liter per minute). * (not measured), ** (not reported).

References

1. Kalisa, E.; Nagato, E. G.; Bizuru, E.; Lee, K. C.; Tang, N.; Pointing, S. B.; Lacap-bugler, D. C. Characterization and Risk Assessment of Atmospheric $PM_{2.5}$ and PM_{10} Particulate-Bound PAHs and NPAHs in Rwanda, Central-East Africa. *Environ. Sci. Technol*. **2018**, 52, 12179–12187
2. Pope, F.D.; Gatari, M. Ng'ang', A. D.; Poynter, A.; Blake, R. Airborne particulate matter monitoring in Kenya using calibrated low-cost sensors. *Atmos .Chem.Phys*. **2018**, 18(20):15403–18.
3. Ouafo-Leumbe, M. R., Galy-Lacaux, C., Liousse, C., Pont, V., Akpo, A., Doumbia, T., & Ekodeck, G. E. Chemical composition and sources of atmospheric aerosols at Djougou (Benin). *Meteo. Atmos. Phys.* **2018**, 1-19.
4. Xu, H.; Léon, J.F.; Liousse, C.; Guinot, B.; Yoboué, V.; Akpo, A. B.; Adon, J.; Ho, K. F.; Ho, S. S. H.; Li, L.; Gardrat, E., Shen, Z., and Cao, J.: Personal exposure to PM2.5 emitted from typical anthropogenic sources in Southern West Africa (SWA): Chemical characteristics and associated health risks, *Atmos. Chem. Phys. Discuss*. **2018**, https://doi.org/10.5194/acp-2018-1060, in review.
5. Djossou, J.; Léon, J. F.; Akpo, A. B.; Liousse, C.; Yoboué, V.; Bedou, M.; & Abbey, M. Mass concentration, optical depth and carbon composition of particulate matter in the major southern West African cities of Cotonou (Benin) and Abidjan (Côte d'Ivoire). *Atmos. Chem. Phys.* **2018**, 18(9), 6275-6291.
6. Omar, S. A. F. A.; & Bouacha, M. I. Study of fine particles PM10 and PM2.5 and three associated heavy metals (Pb, Zn and Cu) in the city of Tiaret, Algeria. *Plant Archives*. **2018**, 18(2), 1879-1883.
7. Terrouche, A.; Ali-Khodja, H.; Kemmouche, A.; Bouziane, M.; Derradji, A., & Charron, A. Identification of sources of atmospheric particulate matter and trace metals in Constantine, Algeria. *Air Quality. Atmos. Health*. **2016**, 9(1), 69-82.
8. Ediagbonya, T. F.; Ukpebor, E. E.; & Okieimen, F. E. Source identification of trace metal in total suspended particulate matter. *Int. J Chem. Sci*. **2015**, 7, 37-45.
9. Kirenga, B.; Meng, Q.; van Gemert, F.; Aanyu-Tukamuhebwa, H.; Chavannes, N.; Katamba, A., Mohsenin, V. The State of Ambient Air Quality in Two Ugandan Cities: A Pilot Cross-Sectional Spatial Assessment. *Int. J. Environ. Res. Public Health*. **2015**, 12, 8075–8091.

10. Bahloul, M.; Chabbi, I.; Dammak, R.; Amdouni, R.; Medhioub, K.; & Azri, C. Geochemical behaviour of PM10 aerosol constituents under the influence of succeeding anticyclonic/cyclonic situations: case of Sfax City, southern Tunisia. *Environ. Monit. Assess*. **2015**, 187(12), 757.
11. Ana, G.; Odeshi,T.A.; Sridhar, M.K.C.; Ige, M.O. Outdoor respirable particulate matter and the lung function status of residents of selected communities in Ibadan, Nigeria. *Perspect. Public Health***. 2014,** 134, 169–175.
12. De Longueville, F.; Hountondji, Y.; Ozer, P.; & Henry, S. The Air quality in African rural environments. Preliminary implications for health: the case of respiratory disease in the Northern Benin. *Water. Air. & Soil. Pollut*. **2014**, 225(11), 2186.
13. Garrison, V. H.; Majewski, M. S.; Konde, L.; Wolf, R. E.; Otto, R. D.; Tsuneoka, Y. Inhalable desert dust, urban emissions, and potentially biotoxic metals in urban Saharan-Sahelian air. *Sci. Total Environ.* **2014**, 500–501, 383–394..
14. Lowenthal, D. H.; Gertler, A. W.; & Labib, M. W. (2014). Particulate matter source apportionment in Cairo: recent measurements and comparison with previous studies. *Int. J. Environ. Sci .Technol.***2014**, 11(3), 657-670.
15. Val, S.; Liousse, C.; Galy-Lacaux, C.; Cachier, H.; Marchand, N.; Badel, A.; Baeza-Squiban, A. Physico-chemical characterization of African urban aerosols (Bamako in Mali and Dakar in Senegal) and their toxic effects in human bronchial epithelial cells: description of a worrying situation. *Part. Fibre Toxicol.* **2013**, 10.
16. De Longueville, F.; Hountondji, Y. C.; Ozer, P.; Marticorena, B.; Chatenet, B.; & Henry, S. Saharan dust impacts on air quality: what are the potential health risks in West Africa?. Human and Ecological Risk Assessment: *An. Int. J.***2013**, 19(6), 1595-1617.
17. Dieme, D.; Cabral-Ndior, M.; Garçon, G.; Verdin, A.; Billet, S.; Cazier, F.; Shirali, P. Relationship between physicochemical characterization and toxicity of fine particulate matter ($PM_{2.5}$) collected in Dakar city (Senegal). *Environ. Res.* **2012**, 113,1–13.
18. Kinney, P. L.; Gichuru, M. G.; Volavka-close, N.; Ngo, N.; Peter, K.; Law, A.; Sclar, E. Traffic Impacts on $PM_{2.5}$ Air Quality in Nairobi, Kenya. *Env. Sci Policy*. **2013**, 14,369–378.
19. Worobiec, A.; Potgieter-Vermaak, S. S.; Berghmans, P.; Winkler, H.; Burger, R., Grieken, R. V. Air particulate emissions in developing countries: A case study in South Africa. *Anal. Lett*. **2011**, 44, 1907–1924.
20. Dionisio, K. L.; Arku, R. E.; Hughes, A. F.; Vallarino, J.; Carmichael, H.; Spengler, J. D.; & Ezzati, M. Air pollution in Accra neighborhoods: spatial, socioeconomic, and temporal patterns. *Environ. Sci .Technol*. **2010**, 2270-2276.
21. Gebre, G., Feleke, Z., & Sahle-Demissie, E. Mass concentrations and elemental composition of urban atmospheric aerosols in Addis Ababa, Ethiopia. *Bull. Chem. Soc. Ethiop*. **2010**, 24(3), 361–73.
22. Mkoma, S.L.; Chi, X.; Maenhaut, W. Characteristics of carbonaceous aerosols in ambient PM10and $PM_{2.5}$ particles in Dar es Salaam, Tanzania. *Sci. Total Environ*. **2010**, 408, 1308–1314.
23. Odhiambo, G. O.; Kinyua, A. M.; Gatebe, C. K.; & Awange, J. Motor vehicles air pollution in Nairobi, Kenya. *Res. J. Environ Earth. Sci*. **2010**, 2(4), 178-187.
24. Zghaid, M.;Noack, Y.; Bounakla, M.; & Benyaich, F. Pollution atmosphérique particulaire dans la ville de Kenitra (Maroc). **2009**, 2268-3798.
25. Boman, J.; Lindén, J.; Thorsson, S.; Holmer, B.; Eliasson, I. A tentative study of urban and suburban fine particles ($PM_{2.5}$) collected in Ouagadougou, Burkina Faso. *X-Ray Spectrom*. **2009**, 38, 354–362.

26. Arku, R. E.; Vallarino, J.; Dionisio, K. L.; Willis, R.; Choi, H.; Wilson, J. G.; Ezzati, M. Characterizing air pollution in two low-income neighborhoods in Accra, Ghana. *Sci. Total Environ.* **2008**, 402, 217–231.
27. Laakso, L.; Laakso, H.; Aalto, P. P.; Keronen, P.; Petäjä, T., Nieminen, T.; & Molefe, M. Basic characteristics of atmospheric particles, trace gases and meteorology in a relatively clean Southern African Savannah environment. *Atmos. Chem. Phys. Discussions*. **2008**, 8(2), 6313-6353.
28. Efe, S. I.; & Efe, A. T. Spatial distribution of particulate matter (PM10) in Warri metropolis, Nigeria. *The Environmentalist*. **2008**, 28(4), 385-394.
29. Van Vliet, E. D. S.; Kinney, P. L. Impacts of roadway emissions on urban particulate matter concentrations in sub-Saharan Africa: New evidence from Nairobi, Kenya. *Environ. Res. Lett*. **2007**, 2, 3–8.
30. Etyemezian, V.; Tesfaye, M.; Yimer, A.; Chow, J. C.; Mesfin, D.; Nega, T.; Wondmagegn, M. Results from a pilot-scale air quality study in Addis Ababa, Ethiopia. *Atmos. Environ.* **2005**, 39, 7849–7860.
31. Engelbrecht, J. P.; Swanepoel, L.; Chow, J. C., Watson, J. G.; & Egami, R. T. PM2.5 and PM10 concentrations from the Qalabotjha low-smoke fuels macro-scale experiment in South Africa. *Environ. Monit. Assess.***2001**, 69(1), 1-15.
